# Solution nuclear magnetic resonance spectroscopy of bacterial outer membrane proteins in natively excreted vesicles using engineered *Escherichia coli*

**DOI:** 10.1002/mbo3.1302

**Published:** 2022-06-26

**Authors:** Mohammed Mouhib, Celestine N. Chi

**Affiliations:** ^1^ Department of Medical Biochemistry and Microbiology Uppsala University Uppsala Sweden; ^2^ Institute of Chemical Sciences and Engineering École Polytechnique Fédérale de Lausanne (EPFL) Lausanne Switzerland

**Keywords:** membrane proteins, nuclear magnetic resonance spectroscopy, outer membrane vesicles

## Abstract

Gaining structural information on membrane proteins in their native lipid environment is a long‐standing challenge in molecular biology. Instead, it is common to employ membrane mimetics, which has been shown to affect protein structure, dynamics, and function severely. Here, we describe the incorporation of a bacterial outer membrane protein (OmpW) into natively excreted membrane vesicles for solution nuclear magnetic resonance (NMR) spectroscopy using a mutant *Escherichia coli* strain with a high outer membrane vesicle (OMV) production rate. We collected NMR spectra from both vesicles containing overexpressed OmpW and vesicles from a control strain to account for the presence of physiologically relevant outer membrane proteins in vesicles and observed distinct resonance signals from OmpW. Due to the increased production of OMVs and the use of non‐uniform sampling techniques we were able to obtain high‐resolution 2D (HSQC) and 3D (HNCO) NMR spectra of our target protein inside its native lipid environment. While this workflow is not yet sufficient to achieve in situ structure determination, our results pave the way for further research on vesicle‐based solution NMR spectroscopy.

## Graphical abstract

We showed a new strategy for producing membrane proteins in native vesicles for structural and biochemical studies, without unfolding and refolding in detergents that often affect the native architecture of these proteins.

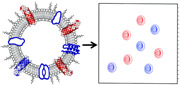

## 1 INTRODUCTION

Biological membranes serve as the diffusion barriers that define cells but need to enable communication with the environment and other organisms beyond cell boundaries. Membrane proteins are crucial for this task and are predicted to account for 20%–30% of all proteins (Wallin & Heijne, 1998). Given their exposure on the surface of cells, it is no surprise that they are being used for vaccine development and targeted by most commercial drugs to date (Overington et al., 2006). For the rational design of these drugs, structural information is essential, but difficult to obtain for membrane proteins due to their hydrophobicity and the inherent need for membrane mimetic environments.

Common membrane mimetic systems are micelles, bicelles, or nanodiscs (Arora et al., 2001; Liang et al., 2014; Raschle et al., 2009). While being widely used in NMR spectroscopy, they are known to impact protein structure and dynamics, which may deteriorate findings from physiological relevant conditions (Chipot et al., 2018; Frey et al., 2017; Mouhib et al., 2021). This is due to two key limitations: (1) the use of detergents throughout any experimental process, and (2) the lack of native membrane architectures. Still, they are important for the solubilization of membrane proteins, which is a prerequisite for the subsequent assembly of lipid bicelles or nanodisc complexes. In structural biology, they are commonly used to extract proteins from membranes or even refold membrane proteins expressed in inclusion bodies, (Liang & Tamm, 2007; Mouhib et al., 2021) which may deteriorate the protein structure further from the native state. While the use of nanodiscs enables a phospholipid bilayer environment for reconstituted proteins, native membrane polarity and glycolipid distribution cannot be reconstructed. Thus, complex interactions of proteins with membrane lipids and carbohydrates are not accurately represented in present biophysical studies across different fields.

There have been attempts to recreate native lipid environments by mixing different phospholipids and later using enzymes such as scramblases (flipases) to cause reversible equilibration of phospholipids in the lipid bilayer (Sharom, 2011). Another approach is the use of a styrene‐maleic acid copolymer to directly disrupt cell membranes from intact cells into smaller fragments, which are later reconstituted into nanodisc‐like particles (Dörr et al., 2014). While these methods are promising and appear applicable in a multitude of studies, they suffer from tedious protocols or low protein yields, which limits their applicability in NMR spectroscopy and other structural studies. Native outer membrane vesicles (OMVs) were previously used as an alternative for biophysical studies such as Cryo‐EM (Zeev‐Ben‐Mordehai et al., 2014), followed by AFM‐based single‐molecule force spectroscopy (Thoma et al., 2018) and solution NMR spectroscopy (Thoma & Burmann, 2020a). While integral membrane proteins proved to be challenging to study using solution NMR spectroscopy (Thoma & Burmann, 2020a), this method enabled the acquisition of high‐resolution NMR spectra from soluble periplasmic proteins in their native environment, which were the focus of follow‐up studies (Thoma & Burmann, 2020a, 2020b, 2022).

Here, we present the use of an engineered *Escherichia coli* strain (ΔnlpI) (McBroom et al., 2006; Ojima et al., 2018; Schwechheimer et al., 2015) for excretion of OMVs to be used for solution NMR spectroscopy of integral membrane proteins, without the use of detergents and avoiding in‐vitro membrane mimetic assembly procedures. The deletion of the outer membrane lipoprotein NlpI causes imbalanced regulation of peptidoglycan degradation and synthesis, leading to a significantly increased rate of OMV production (Schwechheimer et al., 2015). We made use of this mutant to prepare OMV samples with and without overexpressed protein of interest. Coupled with nonuniform sampling techniques, this enabled us to obtain high‐resolution spectra of the *E. coli* outer membrane protein OmpW in its native environment.

## 2 MATERIALS AND METHOD

### 2.1 OmpW expression in the outer membrane

*Escherichia coli* ∆nlpI was obtained from Coli Genetic Stok Center and stored at −80°C on a tissue glycerol stock. For the preparation of electrocompetent cells, an LB‐agar plate containing kanamycin as a selection marker was inoculated with bacteria from the tissue and incubated overnight at 37°C. One colony from the plate was grown in 10 mL SOB‐kanamycin medium (200 rpm, 37°C) overnight. Two drops of this culture were used to inoculate each one of two 1 L Erlenmeyer flasks containing 250 mL SOB each, which were incubated (200 rpm, 37°C) until an OD of about 0.5. The cultures were then chilled on ice for 15 min, and cells were pelleted thereafter (5000 rpm, 10 min). The pellets were re‐suspended in 250 mL ice‐cold 10% glycerol solution and re‐pelleted twice (5000 rpm, 10 min). Finally, the pellet was re‐suspended in residual glycerol and frozen on dry ice. The electro‐competent cells were stored at −80°C until use. *Escherichia coli* ∆nlpI was transformed with a pET21b plasmid containing a gene coding OmpW including a signaling peptide for outer membrane localization by standard electroporation at 1.8 kV. The bacteria were then recovered in 975 µL SOC‐medium (1 h, 37°C, 200 rpm) and plated on LB‐agar plates with ampicillin as a selective marker. For a first overnight culture, 10 mL of LB‐ampicillin medium were inoculated with one colony from the transformation plate (125 mL Erlenmeyer flask, 200 rpm, 37°C). The LB overnight culture was used to inoculate 20 mL of unlabeled M9‐glucose medium, and bacteria were again grown overnight (200 mL Erlenmeyer flask, 200 rpm, 37°C). Five mL of this overnight culture were used to inoculate 400 mL of M9‐glucose medium with 0.5 g/L ^15^NH_4_Cl and/or either 2 g/L 13°C labeled deuterated and nondeuterated glucose or unlabeled glucose for protein expression. Cultures were grown to an OD of about 0.7 and in the case of OmpW‐OMVs induced with 0.5 mM IPTG. Protein expression was carried out for 24 h at 37°C and 200 rpm shaking.

### 2.2 Purification of vesicles with and without overexpressed outer membrane proteins

Expression cultures were pelleted (10 min, 3000 *g*, 4°C). The residual material was precipitated using 400 g/L ammonium sulfate, which was solved by gentle inversion of the containing bottle and incubated for 1.5 h at room temperature. The precipitate was pelleted at 13,000 *g* for 30 min, and obtained pellets were re‐suspended in 10 mL of buffer (20 mM Tris‐HCl, 15% glycerol). Dialysis of the products was performed against 3 L of 20 mM NaPi buffer at a pH of 6.5 using 5 kDa molecular weight cutoff dialysis membranes in two steps. Obtained samples were centrifuged at 3000 *g* for 3 min to remove residual insoluble materials, filtered through 0.45 µM nitrocellulose membranes, and concentrated using 5 kDa cutoff centrifugal filters to a final sample volume of ~250 µL. This final vesicle sample from 400 mL expression volume could be used for 1 NMR sample in a 5 mm Shigemi tube.

### 2.3 NMR spectroscopy

All NMR experiments were performed at 310 K on a Bruker NeoAdvance III 600 MHz spectrometer equipped with a cryogenic triple resonance probe head. Before performing the NMR experiments, all samples were dialyzed into a buffer containing 20 mM sodium phosphate pH 6.5, to which 0.1% NaN_3_ and 10% D_2_O were added. Samples were then transferred to 5 mm Shigemi tubes for NMR spectroscopy. The ^1^H‐^15^N correlation spectra for OmpW‐OMV were recorded using fast NMR methods (Bruker pulse program b_trosyetf3gpsi.2) with a nonuniform sampling rate of 50%, 128 hyper‐complex points in the indirect dimension, and processed with Topspin. For OmpW in nanodiscs, standard 2D ^1^H‐^15^N transverse relaxation optimized spectroscopy‐heteronuclear single quantum coherence (TROSY‐HSQC) with uniform sampling was recorded. The final size of the matrix processed was 4096 (F2) X 2048 (F1) points. The number of scans were varied between 1 k and 256 depending on the protein concentration as determined from absorbance measurements at 280 nm.

### 2.4 Transmission electron microscopy

Samples were prepared by negative staining; A 5‐µL drop of OMV sample in phosphate buffer saline was placed on a formvar and carbon‐coated 200‐mesh copper grid. After 20 s, the excess solution was removed by blotting with filter paper. The sample was then directly stained for 10 s with 2% uranyl acetate and removed with filter paper. Dried grids were then examined by a TEM (FEI Tecnai G2) operated at 80 kV.

### 2.5 Nanodisc assembly

Protein was first expressed in inclusion bodies, purified, and refolded into detergent micelles. Nanodiscs were assembled using phospholipids, OmpW‐micelles, and a truncated ApoA‐I membrane scaffold protein (MSPΔH5). Detailed procedures can be found in Appendix A.

## 3 RESULTS AND DISCUSSION

**Figure 1:**
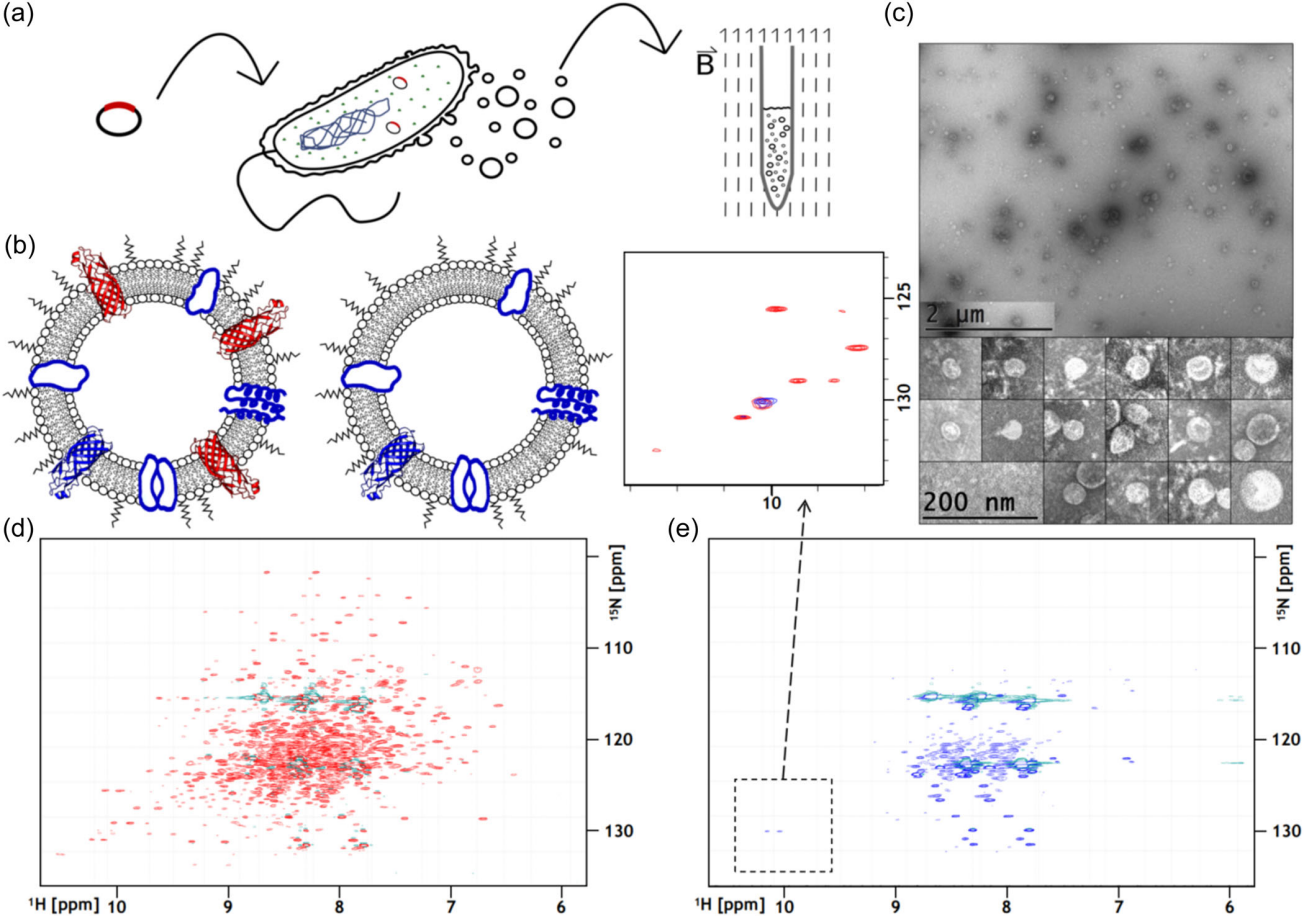
Outer membrane vesicle (OMV) production, characterization, and nuclear magnetic resonance (NMR) spectroscopy. (a) *Escherichia coli* ΔnlpI are transformed with protein of interest coding plasmid and cultured overnight. Vesicles are shed off continuously during bacterial growth and purified from culture supernatants. Recovered vesicles can directly be used for NMR experiments. (b) Schematic representation of OMVs with and without bacterial outer membrane protein (OmpW) overexpression. OmpW is illustrated in red, while physiologically relevant native outer membrane proteins are shown in blue (c) Negative stain transmission electron microscopy (TEM) images of OmpW‐OMVs (d) Transverse relaxation optimized spectroscopy‐heteronuclear single quantum coherence (TROSY‐HSQC) spectra of vesicles with and (e) without OmpW overexpression. The inset shows overlayed spectra showing resonance signals from OmpW‐OMVs (red) and control vesicles (blue).

For the preparation of membrane vesicle samples (Figure 1a–c), we first transformed *E. coli* ΔnlpI with a plasmid encoding OmpW with a signaling sequence for outer membrane localization, and an empty vector as a negative control. After the growth of recombinant strains, OMVs are directly harvested from culture supernatants and used for NMR spectroscopy (Figure 1a). Vesicles were prepared with and without overexpression of OmpW (Figures 1b, A2), to assess whether measured resonance signals can be attributed to OmpW beyond background signals from native proteins and lipids. While vesicles are precipitated using ammonium sulfate and resolved in an appropriate buffer for NMR experiments after excretion from the bacteria, transmission electron microscopy revealed that they maintained their structural integrity (Figure 1c). The purified vesicles displayed varying diameters ranging from approximately 30 to 90 nm. Compared to the diameters of nanodiscs used for NMR spectroscopy, the vesicles are relatively large, with the smallest ones being three times as wide as the average nanodisc (~10 nm).

**Figure 2:**
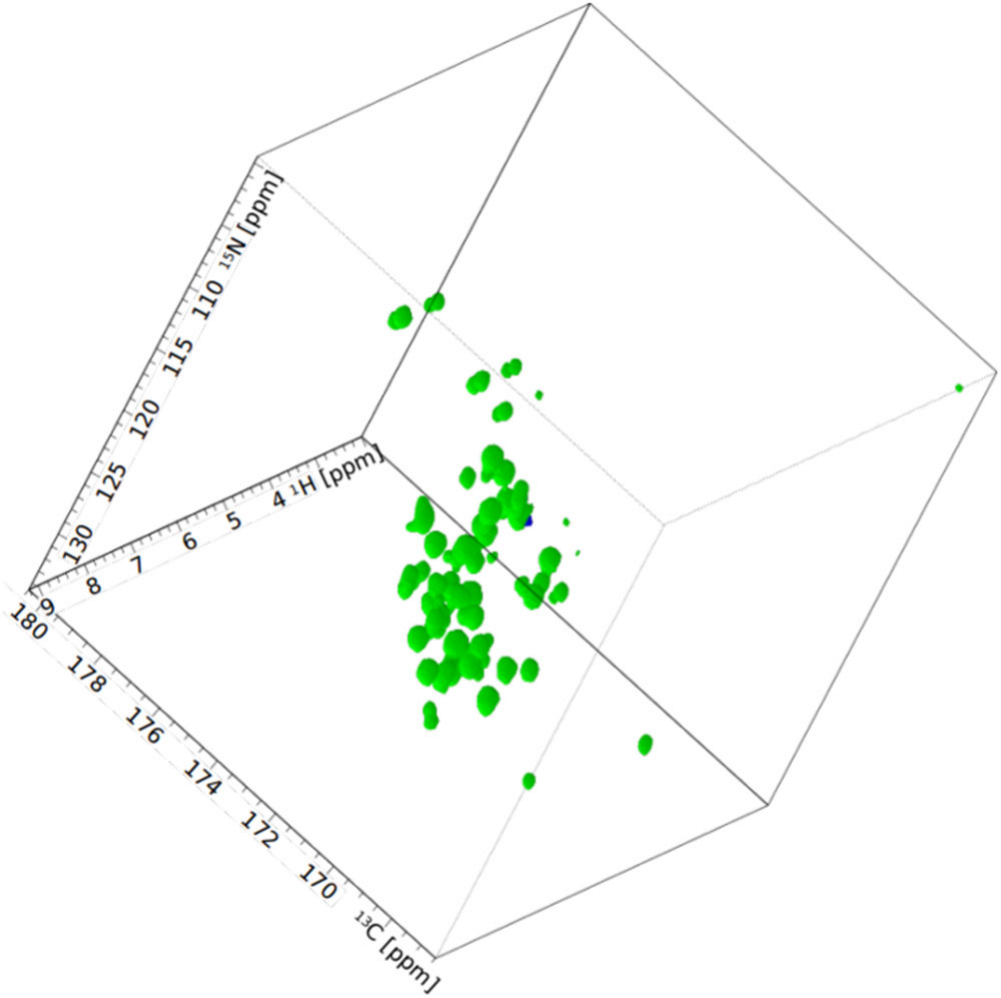
HNCO spectrum of outer membrane protein‐outer membrane vesicles (OmpW‐OMVs). A 3D HNCO type experiment measured on OmpW in purified vesicles. The sample was prepared from a growth medium with 100% D_2_O and enriched in ^13^C and ^15^N isotopes.

The purified vesicles were directly used for solution NMR spectroscopy (Figures 1d,e and 2). The relatively large size of vesicles and associated slow tumbling times would limit their applicability in solution NMR spectroscopy. However, at 310 K and using nonuniform sampling for the acquisition of the NMR data, well‐resolved 2D TROSY‐HSQC spectra could be collected at experimental times of only a few hours (Figure 1d,e). The spectrum from OmpW‐OMVs shows well‐resolved resonance signals throughout the spectral range, with a less dispersed region between 7.5 and 8.5 ppm in the ^1^H‐dimension (Figure 1d). Most of the peaks are missing when OmpW is not overexpressed (Figure 1e), showing that these resonances originate from OmpW beyond the background signal from native *E.coli* proteins. For instance, signals from the five tryptophan side‐chain amide resonances at around 10 ppm in the H‐dimension are well accounted for in the spectrum from OmpW‐OMVs, but not in the background spectrum which only shows one overlapping background peak (10 ppm/130 ppm, H/N) (Figure 1e).

**Figure A1:**
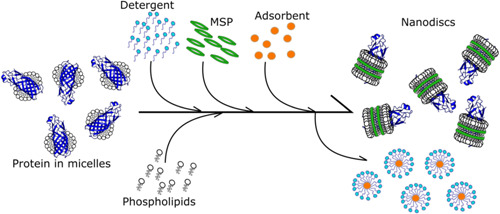
Schematic representation of the nanodisc assembly procedure. The folded protein of interest is dissolved in a detergent. After the addition of bilayer forming lipids and scaffold protein in specific molar ratios, the nanodisc complex is formed. The detergent is removed from the reaction using an adsorbent.

**Figure A2:**
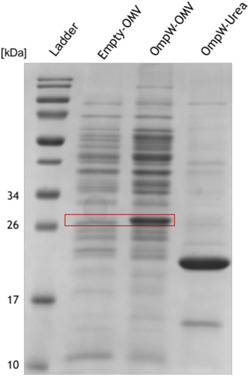
SDS‐PAGE analysis of vesicles and untagged outer membrane protein (OmpW). Samples from vesicles with (OmpW‐ outer membrane vesicle [OMV]) and without (Empty‐OMV) OmpW overexpression, and a sample of untagged OmpW purified in 8 M Urea (OmpW‐Urea) were separated by size, and proteins were stained using Coomassie brilliant blue.

To assess how the in‐vesicle spectrum compares with classic membrane protein in nanodiscs, we assembled 1,2‐dimyristoyl‐sn‐glycero‐3‐phosphocholine (DMPC) 14:1 nanodiscs with reconstituted OmpW. The protein had to be first expressed in inclusion bodies, denatured, purified, and refolded in detergent micelles. These were further processed to replace detergent with phospholipids in a nanodisc complex (Figure A1) (Bayburt et al., 2002; Hagn et al., 2018), which was used for 2D solution NMR experiments. When comparing the ^1^H‐^15^N TROSY‐HSQC spectrum from nanodiscs and OmpW‐OMVs, a clear difference in chemical shift changes can be observed (Figure A2). Those changes further support that artificial environments, such as the DMPC 14:1 nanodiscs, might perturb the native fold and natural properties of embedded membrane proteins (Frey et al., 2017; Mouhib et al., 2021).

**Figure A3:**
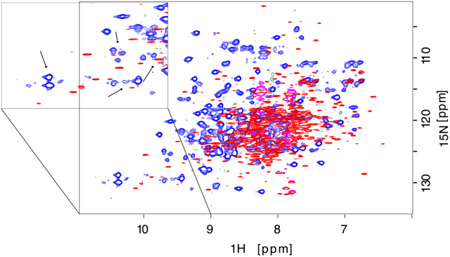
Comparison of outer membrane protein‐outer membrane vesicle (OmpW‐OMV) and nanodisc ^1^H‐^15^N transverse relaxation optimized spectroscopy‐heteronuclear single quantum coherence (TROSY‐HSQC) spectra. Overlay of OmpW‐OMV vesicle (red) spectrum with OmpW 1,2‐dimyristoyl‐sn‐glycero‐3‐phosphocholine (DMPC) 14:1 nanodisc (blue) spectrum. Inset shows the region around 10 ppm H shift where tryptophan epsilon proton shifts are commonly observed.

Lastly, we verified whether the OMVs can be used in other multidimensional NMR experiments (Figure 2). We expressed ^13^C and ^15^N enriched OmpW in a highly deuterated background and performed a 3D HNCO experiment with purified vesicles. The obtained spectrum was of good quality and confirms the applicability of OMVs in solution NMR spectroscopy (Figure A3).

Altogether these results show that OMVs offer a promising perspective in membrane protein NMR spectroscopy and may already be employed in functional studies or to complement data collected using membrane mimetic systems. This approach is limited to outer membrane proteins that can be expressed in *E. coli*, but membrane vesicles have also been reported in Gram‐positive bacteria (Liu et al., 2018), which may be the focus of future NMR studies to expand the range of potential analytes. The performed HSQC and HNCO experiments are highly sensitive, but less sensitive experiments such as HNCACB will be needed for full resonance assignments and nuclear Overhauser effect (NOE)‐based structure determination. While this workflow is not yet sufficient to achieve in situ structure determination, our results pave the way for further research efforts on vesicle‐based solution NMR spectroscopy. Potential bioengineering approaches for the selective production of smaller vesicles and further increases in production rates, as well as efficient size separation of vesicles, may be future stepping stones.

## AUTHOR CONTRIBUTIONS

**Mohammed Mouhib**: conceptualization (equal); data curation (equal); formal analysis (equal); investigation (equal); methodology (equal); writing – original draft (equal); writing – review & editing (equal). **Celestine N. Chi**: conceptualization (equal); formal analysis (equal); funding acquisition (equal); investigation (equal); methodology (equal); project administration (equal); resources (equal); supervision (equal); writing – original draft (equal); writing – review & editing (equal).

## CONFLICT OF INTEREST

None declared.

## ETHICS STATEMENT

None required.

## Figures and Tables

**Figure A4:**
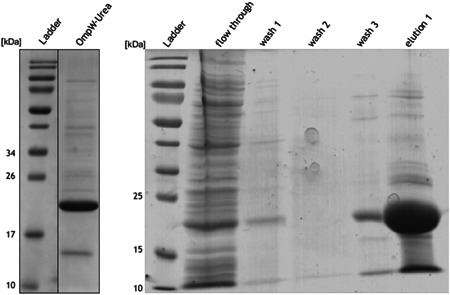
SDS‐PAGE analysis of untagged outer membrane protein (OmpW) and MSPΔH5. SDS‐PAGE analysis of purified OmpW after complete denaturation in Urea (OmpW‐Urea, cropped from Figure A1) and of fractions during Ni^2+^ affinity purification of HIS‐tagged MSPΔH5. The washing steps 1–3 were carried out in buffer, buffer with 50 mM sodium cholate and buffer with 20 mM imidazole, respectively. 0.5 M imidazole was used for elution.

## Data Availability

All data are included in this published article.

## APPENDIX A

**Additional methods**

**Expression of outer membrane protein (OmpW) in inclusion bodies**

For the expression of OmpW in inclusion bodies, a pET‐21b plasmid containing the OmpW gene without outer membrane localization signal was purchased from GenScript. The construct was transformed into *Escherichia coli* OneShot BL21 (DE3) cells (Invitrogen) by standard heat shock transformation. Bacteria were first grown in a pre‐culture of 20 mL M9‐glucose medium (50 µg/mL kanamycin) and incubated overnight (200 rpm, 37°C). This preculture was used to inoculate two 5‐L Erlenmeyer flasks containing 500 mL of deuterated M9‐glucose medium with 0.5 g/L ^15^NH_4_Cl (50 µg/mL kanamycin). At an OD >0.6, the culture was induced with 0.5 mM IPTG and grown for 1 h at 37°C and 3 h at 28°C thereafter. Cultures were then pelleted and cells re‐suspended in 15 mL of buffer (20 mM Tris‐HCl, pH 8, 0.5 M NaCl, 0.5% Triton X‐100).

**OmpW purification and reconstitution in detergent micelles**

A tablet of protease inhibitor (Roche complete mini protease inhibitor, EDTA‐free) was added to the suspension. The mixture was shaken (30 min, 4°C) and the cells were lysed by sonication (35 psi, 6 min). The lysate was then centrifuged (1.5 h, 47,800 *g*, 4°C), and pellets were re‐suspended in 20 mM Tris‐HCl, pH 8, 0.5 M NaCl, and 0.5 v/v Triton X‐100 with further shaking (1 h, 37°C). Subsequently, the suspensions were centrifuged (4°C, 8000 *g*, 35 min), pellets were resuspended (20 mM Tris‐HCl, pH 8, 0.5 M NaCl), and centrifuged again (4°C, 8000 *g*, 35 min), and finally dissolved in 50 mL 20 mM Tris‐HCl, pH 8 with 6 M GdmCl. The supernatants from the last centrifugation step (4°C, 47800 *g*, 30 min) were stored at 4°C.

For refolding, the sample was added slowly (300 µL/min) into a 50‐mL reservoir of buffer (50 mM Tris‐HCl, pH 8, 100 mM NaCl, 500 mM l‐arginine, 0.5 mM DPC) while stirring. After the addition, the mixture was further stirred for 1 h at RT.

The micelles were dialyzed (100 mM NaCl, 20 mM Tris, pH 7.4) and then concentrated using Sartorius centrifugal 10 kDa cut‐off filter (6°C, 4400 *g*).

**Expression and purification of MSPΔH5**

The MSPΔH5 expressing strain was obtained by transforming *E. coli* BL21 pLyS cells with pET28a MSPΔH5, selecting with 50 µg/mL kanamycin and 35 µg/mL chloramphenicol. Transformed cells were cultivated in a 30 mL LB medium containing the same antibiotics (1 h, 37 C, 200 rpm).

Next 5 mL of preculture were used to inoculate each of six 2‐L Erlenmeyer flasks containing 500 mL of LB medium with the same antibiotics as above. After reaching an OD > 0.6, the cultures were induced with 0.5 mM IPTG and grown for 1 h at 37°C and 2 h at 28°C thereafter. Cultures were then pelleted (5000 *g*, 20 min, 4°C), and re‐suspended in 15 mL of buffer A (20 mM Tris‐HCl, pH 8, 0.5 M NaCl, 0.5% Triton X‐100) each.

Two tablets of protease inhibitor (Roche complete mini protease inhibitor, EDTA‐free) were added. After shaking (30 min, RT) the cells were lysed by sonication (35 psi, 6 min). The lysate was then centrifuged (1.5 h, 20000 rpm, 4°C) and filtered (0.2 µm). Afterward, MSPΔH5 was purified by affinity chromatography using a Ni^2+^ column. The washing steps were performed using buffer A (6‐column volumes), buffer A with 50 mM sodium cholate (6‐column volumes), buffer A (4‐column volumes), and finally, buffer A with 20 mM of imidazole (8‐column volumes). The protein was eluted using an imidazole concentration of 0.5 M.

The buffer was exchanged using a PD‐10 column from GE Healthcare. The exchange was performed according to the manufacturer's guidelines. The elution buffer was composed of 20 mM Tris‐HCl, pH 8, 100 mM NaCl, and 50 mM sodium cholate.

**Assembly of OmpW‐MSPΔH5 nanodiscs**

The molar ratio of MSP**Δ**H5: OmpW: DMPC: Na‐cholate was set to 2:1: 80:160. All of the components were mixed in a 7 mL volume and incubated overnight (16 h, 150 rpm, 27°C). After the addition of 1 g/mL Bio‐BeadsTM (Bio‐Rad), the mixture was incubated for 4 h (150 rpm, 27°C). The Bio‐BeadsTM were removed by low‐speed centrifugation. The supernatant was concentrated and applied to a Superdex‐200 column for size exclusion chromatography, using 20 mM NaPi, pH 7.5 with 100 mM NaCl as running buffer. Main fractions were thereafter pooled and dialyzed (5 mM EDTA, 20 mM NaPi, pH 7.5). The sample was then concentrated for use in NMR spectroscopy using a 30 kDa Amicon centrifugal filter. (Figures A1, A2, A3, A4).
